# The First Report to Evaluate Safety of Cyanobacterium *Leptolyngbya* sp. KIOST-1 for Use as a Food Ingredient: Oral Acute Toxicity and Genotoxicity Study

**DOI:** 10.4014/jmb.2007.07013

**Published:** 2020-11-17

**Authors:** Youngdeuk Lee, Taeho Kim, Won-Kyu Lee, Yong-Kyun Ryu, Ji Hyung Kim, Younsik Jeong, Areumi Park, Yeon-Ji Lee, Chulhong Oh, Do-Hyung Kang

**Affiliations:** 1 Jeju Marine Research center, Korea Institute of Ocean Science and Technology, Jeju Special Self-Governing Province 63349, Republic of Korea; 2 Current address: Infectious Disease Research Center, Korea Research Institute of Bioscience and Biotechnology, Daejeon 34141, Republic of Korea

**Keywords:** *Leptolyngbya* sp. KIOST-1, cyanobacterium, food ingredient, genetic toxicity, single-dose oral toxicity

## Abstract

*Leptolyngbya* sp. KIOST-1 (LK1) is a newly isolated cyanobacterium that shows no obvious cytotoxicity and contains high protein content for both human and animal diets. However, only limited information is available on its toxic effects. The purpose of this study was to validate the safety of LK1 powder. Following Organisation for Economic Co-operation and Development (OECD) guidelines, a single-dose oral toxicity test in Sprague Dawley rats was performed. Genotoxicity was assessed using a bacterial reverse mutation test with *Salmonella typhimurium* (strains TA98, TA100, TA1535, and TA1537) and *Escherichia coli* WP2 *uvrA*, an in vitro mammalian chromosome aberration test using Chinese hamster lung cells, and an in vivo mammalian erythrocyte micronucleus test using Hsd:ICR (CD-1) SPF mouse bone marrow. After LK1 administration (2,500 mg/kg), there were no LK1-related body weight changes or necropsy findings. The reverse mutation test showed no increased reverse mutation upon exposure to 5,000 μg/plate of the LK1 powder, the maximum tested amount. The chromosome aberration test and micronucleus assay demonstrated no chromosomal abnormalities and genotoxicity, respectively, in the presence of the LK1 powder. The absence of physiological findings and genetic abnormalities suggests that LK1 powder is appropriate as a candidate biomass to be used as a safe food ingredient.

## Introduction

Cyanobacteria are widely used as a food product or dietary supplement in several countries [1, 2], and these bacteria are considered a source of protein for both humans and animals. There are numerous records of historical usage of cyanobacteria in the human diet [3]. Spirulina is one of the most popular cyanobacteria and has been marketed and consumed as a human food source in more than 70 countries [3]. For example, the local population in Chad has used spirulina as a daily dietary component [4]. In addition to spirulina, several other cyanobacteria have been used as food. *Nostoc commune* is consumed raw, dried, stir-fried, and in soups or it is used as a thickener for other foods in Asia [3]. *Nostoc flagelliforme* is traditionally a part of human diets in China, Mongolia, and South Africa. The safety of N. flagelliforme as a human food source was evaluated in an acute toxicity study, where oral administration of the dried powder of this organism did not cause any adverse effects in rats [5].

People have become more interested in food safety and their well-being in recent years; thus, the demand for functional foods from natural sources has increased [6]. Spirulina is very popular and well-known as a health-promoting natural product [7]. *Arthrospira platensis* has diverse biological activities owing to its high content of protein, essential amino acids, vitamins, beta-carotene and other pigments, minerals, essential fatty acids, and polysaccharides [8]. The blue pigment C-phycocyanin, which is the major phycobiliprotein in cyanobacteria, is a potential chemotherapeutic agent and can induce apoptosis of tumor cells [9, 10]. Phycocyanin possesses several other bioactivities such as antioxidant, anti-inflammatory, and neuroprotective properties [11, 12]. Therefore, cyanobacteria are considered excellent sources of biologically active compounds that might be important for human health as novel pharmaceuticals.

Previously, we isolated Leptolyngbya sp. KIOST-1 (LK1) and its biochemical composition was compared to that of spirulina (*Arthrospira maxima* Cy-23) [13]. The composition of LK1 was similar to that of *A. maxima* Cy-23, with essential amino acids comprising approximately 40% of the cellular components of both cyanobacterial strains [14]. Moreover, no significant cytotoxicity of LK1 was detected, and no putative cyanotoxin biosynthesis genes were found [14]. Therefore, LK1 could be a highly potent protein source for human and animal consumption. Nevertheless, toxicity testing is one of the most important criteria for screening a new microalga before its commercial use. Toxicity testing is necessary not only to determine how safe a test sample is, but also to characterize any toxic effects that it can produce [15]. The aim of the present study was to emphasize the importance of toxicity testing in the process of discovering new microalgae and developing dietary supplements, specifically focusing on LK1. To verify the safety of LK1 as a dietary supplement, a single-dose oral toxicity study was performed in rats and genotoxicity studies were carried out using bacterial reverse mutation and micronucleus assays, as well as a chromosomal aberration assay with a cultured Chinese hamster lung cell line (CHL/IU).

## Materials and Methods

### LK1 Powder Preparation

Culture medium (SOT medium) for the LK1 (KCTC 12347BP) was formulated in distilled water by adding NaHCO_3_ (16.8 g/l), K_2_HPO_4_ (0.50 g/l), NaNO_3_ (2.50 g/l), K_2_SO_4_ (1.00 g/l), NaCl (1.00 g/l), MgSO_4_·7H_2_O (0.20 g/l), CaCl_2_·2H_2_O (0.04 g/l), FeSO_4_·7H_2_O (0.01 g/l), EDTA (0.08 g/l), and Trace Metal Mix A5 (1.0 mL/L). The micronutrient solution contained H_3_BO_3_ (2.86 mg/l), MnSO_4_·5H_2_O (1.81 mg/l), ZnSO_4_·7H_2_O (0.222 mg/l), Na_2_MoO_4_·2H_2_O (0.39 g/l), CuSO_4_·5H_2_O (0.079 g/l), and Co(NO_3_)_2_·6H_2_O (49.4 mg/l). The LK1 was initially inoculated and grown in a 5 L flask for 1 week, transferred to a 200 L photobioreactor, and then grown for 2 weeks in the laboratory. The culture was grown at 28 ± 0.5°C with agitation under fluorescent lights with an illumination intensity of 60 μmol photons/(m^2^·s) and a 12-h light: 12-h dark cycle. For the scale-up culture in a 20-tonne open raceway system (ORS), the strain was cultured in medium formulated in tap water containing the same nutrients as mentioned above under a natural photoperiodic cycle with agitation using a paddlewheel. After growing the strain for 10 days in the ORS, when the concentration of LK1 was > 0.5 g/l, the cultured LK1 samples were harvested from the whole culture medium by using a Tubular Separator (GQLY series) at 15,000 rpm for 3 h. The harvested samples were stored in a deep freezer (Deasan, Korea) at -50°C and then lyophilized using a freeze dryer (Ilshin, Korea) at the Jeju Marine Research Center of the Korea Institute of Ocean Science & Technology (KIOST).

All experiments were conducted at AAALAC-certified ChemOn Inc. (Korea), an institution authorized to perform nonclinical studies under Good Laboratory Practice (GLP) regulations of the Korea Food and Drug Administration (KFDA) and were consistent with Organisation for Economic Co-operation and Development (OECD) guidelines [16].

### Single-Dose Oral Toxicity

**Animal care and maintenance.** The present study was approved by the Institutional Animal Care and Use Committee (IACUC) of ChemOn Inc. (Serial No. 15-R304). Forty-four female and male Sprague Dawley rats (7 weeks old) were obtained from SAMTAKO BIO KOREA (Korea). Sprague Dawley rats are widely used in toxicity studies and a large historical database exists for this species, allowing for the comprehensive interpretation and evaluation of the results of this study. The animals were individually weighed and visually inspected upon receipt and then acclimated under laboratory conditions for 7 days. General clinical observations were made once per day. According to the certificate provided by the supplier, there were no external factors that could have had an effect on the study. The body weight ranges of the rats were 237.59-257.20 g and 173.79-188.88 g upon LK1 powder dosing for the male and female rats, respectively.

The animals were housed at 23 ± 3°C with a relative humidity of 55 ± 15%, 12-h light/12-h dark cycle (from 08:00 to 20:00), 150-300 Lux of luminous intensity, and 10-20 air changes per hour. The animals were fed an irradiation-sterilized pellet diet for lab animals (TEKLAD CERTIFIED IRRADIATED GLOBAL 18% PROTEIN RODENT DIET, 2918C, Harlan Laboratories, USA) *ad libitum*. According to the diet composition and contaminant certification provided by the supplier, there was no diet-related factor that could have affected the results of this study. No more than three animals were housed in a stainless steel cage (W 215 mm × L 355 mm × H 200 mm). Water was checked daily and water bottles were replaced more than once per week.

**LK1 administration.** No mortality and clinical signs were observed in a pilot test using doses of 625, 1,250, and 2,500 mg/kg to one animal per group. Based on the above result, these doses were selected for further study. A vehicle control group, orally administered sterile water only, was also established. Healthy animals selected after the acclimation period were weighed and their body weights were ranked. Then, the animals with body weights close to the average were selected and randomly distributed into four groups, each composed of five male and five female rats. After overnight fasting, the dose volume was calculated in order to administer 20 ml/kg based on the fasted body weight. LK1 was then directly administered into the stomach by gavage using a syringe tube with a feeding needle. Three to four hours after dosing, food was again provided *ad libitum*.

**Observations and examinations**. The animals were observed continuously during the first hour and then hourly until 6 h after dosing. The day of dosing was designated as Day 1. Every animal was observed daily for clinical signs from Day 2 to Day 15, and the type and severity of any signs and the date of observation were recorded. All animals were weighed on Day 1 (before administration) and on Days 2, 4, 8, and 15. On Day 15, all animals were anesthetized by CO_2_ inhalation, and after laparotomy, were euthanized by exsanguination from the posterior vena cava and abdominal aorta. All organs were visually examined.

### Bacterial Reverse Mutation Assay

Positive controls were selected from those recommended in the OECD guideline TG471. 2-Aminoanthracene (2-AA) and benzo[a]pyrene (B[a]P) were used as positive controls for metabolic activation. Sodium azide (SA), 2-nitrofluorene (2-NF), 4-nitroquinoline-1-oxide (4NQO), and Acridine Mutagen ICR 191 (ICR-191) were used as positive controls without metabolic activation. All positive controls were purchased from Sigma-Aldrich (USA). The S9 mix (5% v/v) was prepared using 50 μl of Aroclor 1254-induced rat liver S9 (Molecular Toxicology Inc., USA) supplemented with cofactor-I, consisting of 8 μmol MgCl_2_·6H_2_O, 33 μmol KCl, 5 μmol glucose 6-phosphate, 4 μmol NADH, and 100 μmol sodium phosphate buffer (pH 7.5).

The histidine auxotrophic strains of *Salmonella* typhimurium, TA100, TA1535, TA98, and TA1537 [17], and the tryptophan auxotrophic strain of *Escherichia coli*, WP2 *uvrA* [18], were used. TA100, TA1535, and WP2 *uvrA* were used to detect base-pair substitution mutagens, whereas TA98 and TA1537 were used to detect frame-shift mutagens. All the test strains used were obtained from Molecular Toxicology Inc. These test strains are among those recommended by the test guidelines of the Ministry of Food and Drug Safety and have been shown to be sensitive to the mutagenic activity of a wide range of chemical classes.

To set the proper treatment concentration of LK1, a range-finding test was carried out. In the range-finding test, the test strains were treated with eight levels of LK1, ranging from 5 to 5,000 μg/plate, and the treatment mixtures and plates were checked for the absence of precipitation and cytotoxicity. At the time of colony counting, precipitation was observed at 5,000 μg/plate, and no cytotoxicity was observed in the plates at the time of plate scoring. There were no significant increases in revertants per plate at any dose level in any of the test strains. Therefore, the treatment ranges (15, 50, 150, 500, 1,500, and 5,000 μg/plate) were set based on the results of the range-finding test conducted on the LK1 powder using the five test strains in the presence (with S9) and absence (without S9) of metabolic activation, with one plate per mass. The test strains were exposed to the LK1 powder using the direct plate incorporation method. A small amount of bacterial growth in each master plate was transferred to a flask containing 20 ml of liquid medium (2.5% Oxoid Nutrient Broth No. 2). The inoculated flasks were incubated for 10 h in a shaker/incubator (37 ± 2°C, 120 rpm). The overnight cultures were removed from incubation and viable cell counts were determined by optical density (OD) at 600 nm. The cultures were stored in a refrigerator until use. For the plating assay, the following was added to each sterile culture tube containing 2 ml of top agar held at 45 ± 2°C in a dry bath: 0.5 ml of S9 mix (or sodium phosphate buffer, pH 7.4, for the non-activating plates), 0.1 ml of bacterial culture, and 0.1 ml of LK1 powder. The contents were vortexed for 2-3 s and overlaid on the surface of the bottom agar. The negative control plates were treated with 0.1 ml of vehicle (sterile distilled water) instead of LK1 powder. The positive control plates were treated with the positive control compounds using the same method. The sterility of the highest concentration of LK1 solution was verified by plating a 0.1-ml aliquot (mixed with 2 ml of top agar) on the minimal glucose agar. The S9 mix was also tested for sterility by plating 0.5 ml using the same method. After the top agar solidified, the plates were inverted and incubated at 37 ± 2°C for 50 ± 2 h, and then, the revertant colonies were counted by unaided eye.

Cytotoxicity was defined as a clearing or diminution of the background lawn that was accompanied by a substantial reduction in the number of revertants per plate and the presence of microcolonies. A reduction in the number of revertants was assessed if the number of revertants per plate was less than 50% that of the negative control or if there was a reversal of an increasing trend in the number of colonies.

### In Vitro Chromosome Aberration Test in Chinese Hamster Lung Cells

The CHL/IU cell line (American Type Culture Collection, USA), originally derived from the lung of a female Chinese hamster, was used. This cell line has been demonstrated to be sensitive to the clastogenic activity of a variety of chemical agents. The cells were thawed and cultured at least 7 days prior to experimentation. The cells were grown in Minimum Essential Medium (Gibco-BRL, UK). A 440-ml aliquot of this medium was supplemented with 5 ml of GlutaMax I Supplement, 5 ml of penicillin streptomycin (Gibco-BRL), and 50 ml of fetal bovine serum (Gibco-BRL). The cultures were incubated in a humidified incubator at 37 ± 1°C in an atmosphere of 5% CO_2_ in air and were subcultured every 2-3 days using 0.1% trypsin solution.

A preliminary study was performed, using one culture per concentration, to determine the highest concentration required for this analysis in both the presence and absence of metabolic activation. The S9 mix was used at 20% for metabolic activation. B[a]P and 4NQO were used as positive controls in the presence (with S9) and absence (without S9) of metabolic activation, respectively. The cells were removed from the flasks and counted ~24 h after the start of treatment. Based on the results of this preliminary experiment, the concentration at which the relative increase in cell counts (RICC) was less than 50% was set as the maximum concentration.

Three series of cultures for treatment were established as follows. Treatment series-1: presence of S9 mix, 6-h treatment/ 18-h recovery (0, 87.5, 175, or 350 μg/ml of LK1); Treatment series-2: absence of S9 mix, 6-h treatment/ 18-h recovery (0, 250, 500, or 1,000 μg/ml of LK1); Treatment series-3: absence of S9 mix, 22-h treatment/ 2-h recovery (0, 250, 500, or 1,000 μg/ml of LK1).

Before treatment, the old medium was replaced with fresh medium. LK1 was added to each flask at least 1 h after the medium change. The treatment mixtures were removed after 6 h (6 h treatment + 18 h recovery with/without S9) and after 22 h 22 h treatment + 2 h recovery without S9) and the cell monolayers were washed once with Ca^2+^-and Mg^2+^-free Dulbecco’s phosphate-buffered saline. For the recovery period, the cells were given fresh medium and incubated. Colchicine solution was then added to each culture (final concentration of 1 μM) and incubated for 2 h for mitotic arrest. The mitotic cells were detached by gentle shaking. The cell-containing medium was centrifuged at 200 ×*g* for 5 min, and the cell pellets were resuspended in 75 mM potassium chloride solution for hypotonic treatment. Then, the cells were fixed with methanol:glacial acetic acid (3:1 v/v) three times and mounted on slides by the air-drying method. The slides were stained with 5% Giemsa solution. After harvesting the mitotic cells, the remaining cell monolayer in each flask was trypsinized and counted to calculate the RICC.

Morphological classification and counting of chromosomal aberrations was carried out according to the principles of the Atlas of chromosome aberration by chemicals [19]. The slides were coded for blinded reading and were examined under an optical microscope at 100× magnification. One hundred well-spread metaphases per slide were evaluated. Metaphases with 23-27 centromeres were evaluated for structural chromosomal aberrations, which were classified into chromosome-type deletion/exchange and chromatid-type deletion/exchange. The frequency of metaphases with aberrations for each culture, both inclusive and exclusive of gaps, was calculated. A metaphase with more than 10 aberrations (multiple aberrations including gaps) or with chromosome fragmentation was classified as “other” and counted as one aberration. Regardless of the presence of aberrations, 150 metaphases/culture were examined to determine the frequencies of diploidy (23-36 centromeres), polyploidy (≥ 37 centromeres), and endoreduplication.

A metaphase with at least one structural chromosome aberration was classified as an aberrant cell. The number of aberrant cells, except those having only gaps, was subjected to statistical analyses. Fisher's exact test was used to compare the frequency of aberrant cells between the negative control and treated groups. The significance level was set at *p* < 0.05. The sum of the metaphases with ≥ 37 centromeres and metaphases with endoreduplication was evaluated in the same way as those with structural aberrations. The result was considered positive if there was a concentration-dependent increase in the number of aberrant metaphases or a reproducible increase in at least one concentration level. However, statistical significance was not the only determining factor for a positive response. The biological relevance, the frequency of aberrant metaphases, and cytotoxicity were also considered.

### In Vivo Micronucleus Test

The oral administration of LK1 at a dose of 2,500 mg/kg did not produce overt toxicity in rats. Thus, 2,500 mg/kg was selected as the maximum dose. Cyclophosphamide monohydrate (CPA) (Sigma-Aldrich Co.) was used as the positive control, which is recommended in OECD guideline TG474. Hsd:ICR (CD-1) SPF mice (Koatech, Korea) were visually inspected upon arrival and acclimatized for 7 days. The animal laboratory conditions, diet, and water were the same as those described above in the single-dose oral toxicity study using rats. To determine optimal doses in a preliminary study, LK1 was administered (20 ml/(kg·day)) orally to three male and three female mice once daily for 2 days at 625, 1,250, or 2,500 mg/(kg·day). Observations were conducted for 4 days including the treatment day. All animals showed compound-colored stool on Days 2 and 3, but no abnormality was observed by Day 4.

Accordingly, there were three dose groups that were administered LK1 (0 mg/(kg·day), 1,250 mg/(kg·day), and 2,500 mg/(kg·day)) once daily for 2 days, with six males per group. The positive control CPA (70 mg/(kg·day)) was intraperitoneally injected once on Day 2. The mice were euthanized by CO_2_ gas inhalation 24 h after the last administration. Bone marrow specimens were collected from each euthanized mouse using a 23G syringe and suspended in fetal bovine serum (HyClone; GE Healthcare Life Sciences, USA) [20]. The cell suspensions were centrifuged, smeared on slides, dried, and fixed in methanol for 5 min. Two slides were prepared per mouse. The mounted specimens were stained with acridine orange solution (0.05%) diluted in Sorensen's buffer (1:4 v/v, pH 6.8) and observed under a fluorescence microscope at 400× magnification. The numbers of micronucleated polychromatic erythrocytes (MNPCEs) were counted among 4000 polychromatic erythrocytes (PCEs) per animal, and the MNPCE frequency was calculated as the average number of MNPCEs per 4000 PCEs. Morphological examinations were conducted according to the method described by Hayashi *et al*. [21] and were regarded as valid when the following conditions were satisfied. 1) More than five animals were alive at autopsy in every dose group. 2) The average of the PCE/red blood cell (RBC) ratios in the control and dose groups was more than 20% of the value in the negative control group. 3) Among 4000 PCEs, the average MNPCE frequencies in the positive and negative control groups were similar within the range of historical control data, and the positive control result was significantly higher than the negative control result.

### Statistical Analysis

Statistical analysis was performed using SPSS Statistics 22 for Medical Science, with a significance level of *p* < 0.05. Parametric multiple comparisons were used between groups. Body weight data were assumed to be normally distributed and were analyzed by one-way ANOVA. The nonparametric Kruskal-Wallis H test using ranked data was performed for micronucleus frequency and showed no significant differences when compared with the negative control. The significance rates of the positive and negative control data were verified by Mann-Whitney U tests. Parametric one-way ANOVA was utilized to compare PCE/RBC ratios and body weights, assuming equal variance, which was verified by Levene’s test. Significant differences were identified between the maximum dose group and the negative control group by ANOVA and Duncan's multiple range tests. The treatment was regarded as cytotoxic when the average PCE/RBC ratio in the dose groups was significantly reduced. The mutagenicity was regarded as positive when the MNPCE frequency was statistically significant and increased dose-dependently in the treated groups, or when it showed a reproducible increase in one or more dose groups. All figures were created using GraphPad Prism 8.2.1 software (version 8.2.1, GraphPad Software Inc., USA).

## Results

### Single-Dose Toxicity Study

**Fig. 1:**
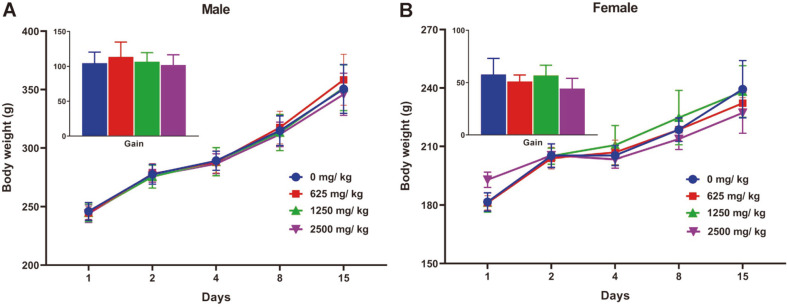
Body weight changes and overall gains (inset) in LK1-administered male (A) and female (B) rats during the experimental period. LK1, *Leptolyngbya* sp. KIOST-1. Data are presented as means ± standard deviations (*n* = 5).

A reddish tear was observed with two male rats in the 2,500 mg/kg group on the dosing day, and compound-colored stool was observed with all animals administered LK1 powder on Day 2. In the single-dose toxicity study, there was no mortality, no LK1 powder-related body weight changes (Fig. 1), and no necropsy findings.

### Bacterial Reverse Mutation Assay

**Fig. 2:**
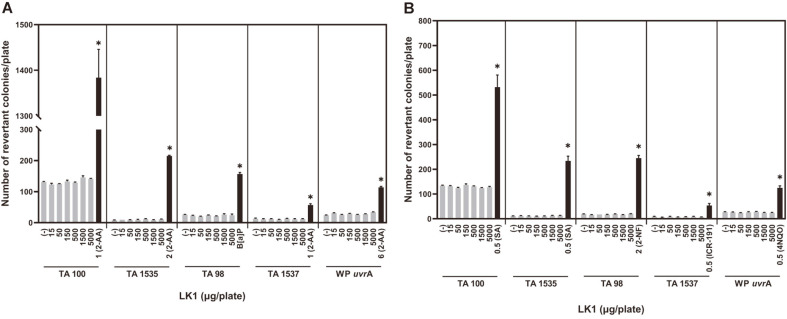
Effects of LK1 on bacterial reverse mutation with S9 metabolic activation (A) and without S9 metabolic activation (B). The graphs show the number of reverse-mutated colonies that grew on agar plates (mean + standard deviation, *n* = 3). * Significantly different at *p* < 0.05 compared with corresponding negative control. Negative controls are indicated with (-). 2-Aminoanthracene (2-AA), benzo[a]pyrene (B[a]P), sodium azide (SA), 2-nitrofluorene (2-NF), Acridine Mutagen ICR 191 (ICR-191), and 4-nitroquinoline-1-oxide (4NQO) were used as positive controls. LK1, *Leptolyngbya* sp. KIOST-1.

The bacterial reverse mutation assay results are shown in Fig. 2. There was no microbial contamination on any of the plates used for sterility testing of LK1 and the S9 mix. There was no decrease in revertants or cytotoxicity in all S. typhimurium strains at all tested masses of LK1. No mass-related increase in the numbers of revertants per plate was observed with all strains. With WP2 *uvrA*, there was neither an increase in colonies nor cytotoxicity at any amount of LK1. The mean number of revertants of the positive control for each test strain was clearly greater than the mean number of revertants of the corresponding negative control. The viable cell counts of the test strains were 0.70-0.97 × 10^9^ (TA strains) and 1.43 × 10^9^ (*E. coli*) CFU/ml, and at least 0.5 × 10^8^ CFU of bacteria/plate were plated.

### In Vitro Chromosome Aberration Test in Chinese Hamster Lung Cells

The chromosome aberration test results are shown in Table 1. Precipitation of LK1 powder was observed at all tested concentrations in the presence and absence of the S9 mix. In the presence of S9, the mean frequency of metaphases with structural aberrations was 0.0 in the negative control and with all concentrations of LK1 (Table 1). In the 6-h treatment group in the absence of S9, the mean frequencies of metaphases with structural aberrations were 0.5, 0.0, 0.5, and 2.0 in the negative control, 250, 500, and 1,000 μg/ml LK1 groups, respectively. In the 22-h treatment in the absence of S9, the mean frequencies of metaphases with structural aberrations were 0.0, 0.5, 0.5, and 0.0 in the negative control, 250, 500, and 1,000 μg/ml LK1 groups, respectively. There were no statistically significant increases at any LK1 concentration in the presence and absence of the S9 mix. In contrast, in the positive control, there was a statistically significant increase in the frequency of aberrant metaphases compared to that in the negative control.

### In Vivo Micronucleus Test

The MNPCE frequencies observed in 4000 PCEs per animal were 2.67, 2.83, 2.0, and 2.0 for the negative control, 625, 1,250, and 2,500 mg/(kg·day) groups, respectively. There was no statistically significant increase in MNPCE frequencies in the test groups compared to that in the negative control group. In contrast, in the positive control group, the MNPCE frequency was 90.83, which was significantly higher (*p* < 0.01) than the frequency in the negative control group. The ratios of PCE:RBC, known as a cytotoxicity indicators, were 0.57, 0.59, 0.58, and 0.59 for the negative control, 625, 1,250, and 2,500 mg/(kg·day) groups, respectively, which were not significantly different. Meanwhile, the PCE:RBC ratio was 0.49 in the positive control and was significantly decreased (*p* < 0.01) compared to the negative control ratio. The micronucleus assay results are shown in Table 2.

## Discussion

The present study was conducted to investigate the potential acute toxicity of LK1 in Sprague Dawley rats after a single oral administration. During the experimental period, a reddish tear on the dosing day and compound-colored stool from all animals 2 days after administration were observed. However, there was no mortality. The compound-colored stool was considered an effect of excretion of LK1 and its metabolites. Because the reddish tears were observed with two animals in the high-dose group, they were considered an LK1-related change. These manifestations did not affect body weight increases and were therefore considered temporary, as typically found in other animal experiments. Furthermore, there were no LK1-related body weight changes and necropsy findings. Based on the above results, the approximate lethal dose of LK1 is considered higher than 2,500 mg/kg when orally administered to Sprague Dawley rats.

The basic principle of toxicity testing is first to determine the direct effect of the test substance on the test animal, and second to extrapolate the risk of exposure of the substance from the test animal to humans. In this study, the maximum dose administered to the experimental animals (2,500 mg/kg) represents the highest concentration of the range suggested by the KFDA. In the KFDA and OECD guidelines, the recommended highest dose of test materials is 2,000 mg/kg; they also recommend that the dosage volume be below 20 ml/kg for single-dose toxicity studies in mice. In the present study, 2,500 mg/kg was selected as the highest dose, which is higher than the recommended highest dosage for test materials. Previous studies using oral administration of environmentally relevant doses of cyanobacterial biomasses containing microcystins demonstrated sublethal effects and high-dose toxicity [22]. However, to the best of our knowledge, there has been no report on the investigation of acute and sublethal toxicity owing to spirulina.

For all of the test strains, in the presence and absence of the S9 mix, there were no significant increases in the number of revertants per plate at any LK1 amount in the bacterial reverse mutation test. There was no statistically significant increase in the frequency of aberrant metaphases with structural and/or numerical aberrations, regardless of the treatment regimen, at all tested concentrations of LK1. Additionally, there was no significant increase in MNPCE frequencies in the test groups compared with that in the control group. All criteria for a valid assay were met, and all results of the genotoxicity testing failed to meet the criteria for positivity. Therefore, LK1 is not considered to induce reverse mutations, chromosomal aberrations, and micronuclei in mouse bone marrow cells under the present test conditions.

Previous reports of in vitro experiments or experiments with laboratory animals suggest that cyanobacteria are an excellent source of biologically active compounds that are beneficial for human health as novel pharmaceuticals. However, several studies have shown that cyanobacteria produce a number of hepatotoxins and neurotoxins such as microcystins, anatoxins, and saxitoxins [23, 24]. Nevertheless, *Leptolyngbya* species are generally known to produce no cyanotoxins [25]. In our previous study, no significant cytotoxicity of LK1 was detected, and the genome of LK1 does not contain cyanotoxin-related genes encoding, for example, anatoxin-a, anatoxin-a(S), β-methylamino-L-alanine (or BMAA), cylindrospermopsin, microcystins, lyngbyatoxin-a, nodularins, and saxitoxins. This genomic information reveals the potential of LK1, and the absence of genotoxicity in this study strongly supports the use of LK1, for alimentary purposes [13, 14].

In conclusion, our results suggest that LK1 does not cause acute toxicity in experimental animals. To establish safety information on LK1 powder, we will conduct additional toxicity studies in the future, including repeated oral toxicity studies, since these reflect how most individuals consume LK1 powder over the long term. The toxicity tests carried out in this study were only short-term, but they included monitoring of physiological parameters and abnormalities and generated accurate data to detect both symptoms and genetic abnormalities. Therefore, the toxicity test results presented herein are likely to be essential data used in the food industry.

## Figures and Tables

**Table 1:** Treatment group organization and summary of chromosome aberration test in Chinese hamster lung cells with LK1.

Condition	Conc. (μg/ml)	Aberrations						PP+ER		No. aberrant metaphase			RICC (%)
		Cs type		Ct type		Other	Gaps	No.	Decision	+Gaps	-Gaps	Decision	
		
		csb	cse	ctb	cte					No.	No.		
6-18	0	0.0	0.0	0.0	0.0	0.0	0.0	0.0	Negative	0.0	0.0	Negative	100
(h)	87.5	0.0	0.0	0.0	0.0	0.0	0.0	0.0	Negative	0.0	0.0	Negative	94
	175	0.0	0.0	0.0	0.0	0.0	0.5	0.0	Negative	0.5	0.0	Negative	88
S9 mix (+)	350 P	0.0	0.0	0.0	0.0	0.0	0.0	0.0	Negative	0.0	0.0	Negative	49
	B[a]P (20)	0.0	0.5	10.5	22.5	0.0	3.0	0.0	Negative	23.5	23.5^**^	Positive	51
6-18	0	0.0	0.0	0.5	0.0	0.0	0.0	0.0	Negative	0.5	0.5	Negative	100
(h)	250	0.0	0.0	0.0	0.0	0.0	0.0	0.0	Negative	0.0	0.0	Negative	91
	500	0.0	0.0	0.5	0.0	0.0	0.0	0.0	Negative	0.5	0.5	Negative	68
S9 mix (-)	1000	0.0	0.0	2.0	0.0	0.0	1.0	0.0	Negative	3.0	2.0	Negative	46
	4NQO (0.4)	0.0	0.5	4.5	10.5	3.0	1.0	0.0	Negative	11.5	11.0^**^	Positive	66
22-2	0	0.0	0.0	0.0	0.0	0.0	0.0	0.0	Negative	0.0	0.0	Negative	100
(h)	250	0.0	0.0	0.5	0.0	0.0	1.0	0.0	Negative	1.5	0.5	Negative	100
	500	0.0	0.0	0.5	0.0	0.0	0.0	0.0	Negative	0.5	0.5	Negative	72
S9 mix (-)	1000	0.0	0.0	0.0	0.0	0.0	0.5	0.0	Negative	0.5	0.0	Negative	43
	4NQO (0.4)	0.0	1.0	7.0	10.0	3.0	2.5	0.0	Negative	19.0	18.0^**^	Positive	64

Numbers of cells examined, aberrations, PP+ER, and metaphases shown are mean values. Benzo[a]pyrene (B[a]P) and 4- nitroquinoline-1-oxide (4NQO) were used as positive controls in the presence and absence of S9, respectively. PP, polyploid; ER, endoreduplication; cd, chromosome; ct, chromatid; csb, chromosome-type deletion; cse, chromosome-type exchange; ctb, chromatid-type deletion; cte, chromatid-type exchange; other, metaphases with more than 10 aberrations (including gaps) or with chromosome fragmentation. **Significantly different from the negative control at *p* < 0.01 (Fisher’s exact test). LK1, *Leptolyngbya* sp. KIOST-1; RICC, relative increase in cell counts

**Table 2:** Micronucleus assay results.

Dose (mg/(kg·day))	Body weights (g) at the time of			MNPCE/4000 PCE (Mean ± SD)	PCE:RBC Ratio (Mean ± SD)	% Control

	1^st^ Admin	2^nd^ Admin	Euthanasia			
0	34.67 ± 1.45	34.30 ± 1.50	34.96 ± 1.34	2.67 ± 1.51	0.57 ± 0.04	100
625	34.82 ± 1.13	34.70 ± 1.32	34.60 ± 1.23	2.83 ± 2.14	0.59 ± 0.02	103
1250	34.61 ± 1.25	34.38 ± 1.36	34.51 ± 1.23	2.00 ± 1.10	0.58 ± 0.04	100
2500	34.83 ± 1.38	34.61 ± 1.05	34.17 ± 1.23	2.00 ± 0.89	0.59 ± 0.02	102
CPA70	34.43 ± 1.50	34.83 ± 1.60	34.54 ± 2.05	90.83 ± 24.04^**^	0.49 ± 0.02^**^	85

Body weights of mice, micronucleus observations, and PCE:RBC ratios are shown. LK1 was orally administered to mice for two consecutive days. CPA was intraperitoneally administered to mice once on the 2^nd^ Admin day. Bone marrow smears were prepared ~24 h after the final administration. The number of MNPCE (micronucleated polychromatic erythrocytes) in 4000 PCE (polychromatic erythrocytes) was analyzed. RBC, red blood cells (polychromatic erythrocyte + normochromatic erythrocyte); CPA, cyclophosphamide monohydrate (positive control); LK1, *Leptolyngbya* sp. KIOST-1; SD, standard deviation. **Significantly different from the negative control group (0 mg/(kg·day)) at *p* < 0.01.
